# The number of neutral mutants in an expanding Luria-Delbrück population is approximately Fréchet

**DOI:** 10.12688/f1000research.127469.1

**Published:** 2022-11-04

**Authors:** Steven A. Frank

**Affiliations:** 1 Department of Ecology and Evolutionary Biology, University of California, Irvine, CA, 92697-2525, USA

**Keywords:** Population genetics, probability distributions, extreme value distributions

## Abstract

**Background:** A growing population of cells accumulates mutations. A single mutation early in the growth process carries forward to all descendant cells, causing the final population to have a lot of mutant cells. When the first mutation happens later in growth, the final population typically has fewer mutants. The number of mutant cells in the final population follows the Luria-Delbrück distribution. The mathematical form of the distribution is known only from its probability generating function. For larger populations of cells, one typically uses computer simulations to estimate the distribution.

**Methods: **This article searches for a simple approximation of the Luria-Delbrück distribution, with an explicit mathematical form that can be used easily in calculations.

**Results:** The Fréchet distribution provides a good approximation for the Luria-Delbrück distribution for neutral mutations, which do not cause a growth rate change relative to the original cells.

**Conclusions:** The Fréchet distribution apparently provides a good match through its description of extreme value problems for multiplicative processes such as exponential growth.

Suppose a single cell expands exponentially to a population of size *N*, with a mutation rate of *u* per cell division. The number of mutant cells, *m*, in the final population depends on the number of mutations that occur and when those mutations occur. For example, a single mutation in the final round of cell division is limited to one cell. By contrast, a single mutation transmitted to one of the daughters in the first cellular division may occur in approximately one-half of the final population.

The distribution of the number mutants, *m*, is known as the Luria–Delbrück distribution ^1^. That distribution is widely used to estimate the mutation rate. The distribution also arises when studying the amount of mutational mosaicism within multicellular individuals ^2– 4^.

Currently, for experiments with a small number of mutational events, one typically calculates the distribution with a probability generating function ^5, 6^. However, that approach becomes numerically inaccurate for larger numbers of mutational events, in which case the distribution is calculated by computer simulation.

This article shows that the Fréchet distribution provides a good approximation for the number of neutral mutants. In particular, the probability that the number of mutants, *m*, is less than *z* is approximately


$$F(z)=\text{Prob}(m<z)=\exp\left(-{\left(\frac{z-\beta }{s}\right)}^{-\alpha }\right),\;(1)$$

in which exp( *z*) = *e ^z^* is the exponential function. The probability of being in the upper tail, *m > z*, is 1 *− F*( *z*). The three parameters set the shape, *α*, the scale, *s*, and the minimum value, *β*, such that *z*, *m > β*.

This form of the Fréchet distribution has three parameters. I found that the following parameterization matches closely the Luria–Delbrück process for neutral mutations


$$\begin{array}{c} \alpha =e/2\\ s=eNu\\ \beta =Nu\log\left(Nu\;e^{-(1+\alpha )}\right) \end{array}$$

in which *e* is the base of the natural logarithm. This parameterization depends on the single parameter, *Nu*, the final population size times the mutation rate.

Figure 1 shows the good fit. Two aspects of mismatch occur. First, the number of mutants is discrete, whereas the Fréchet is continuous. As *Nu* declines to one, significant amounts of probability mass concentrate at particular mutant number values, causing discrepancy between the distributions. Nonetheless, the Fréchet remains a good approximation.

**Figure 1.**
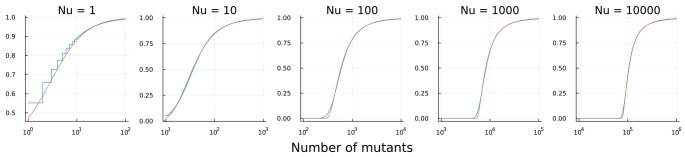
Cumulative distribution of the number of neutral mutants in an expanding population. Each population begins with one cell and grows to *N* cells. Mutation occurs at rate *u*. Blue curves show the distribution from a computer simulation using the simu.cultures command of the R package rSalvador ^7^. Orange curves show the Fréchet distribution in Equation 1. In rSalvador, I used sample sizes of 10 ^6^ or 10 ^7^, values of *Nu* varying as shown above the plots, and values of *N* ranging from 10 ^6^ to 10 ^10^. The Julia software code to produce this figure is available from Zenodo ^8^. The input data for calculating the empirical Luria–Delbrück CDF is also available from Zenodo ^9^.

Second, the lower tail of the Luria–Delbrück process spreads to lower values than the Fréchet. One can see this mismatch most clearly in the figure for *Nu ≥* 100.

This mismatch may occur because the Luria–Delbrück process transitions from a highly stochastic process in earlier cellular generations to a nearly deterministic accumulation of mutations in later cellular generations, when the larger population size reduces the coefficient of variation in the number of new mutations. The Fréchet applies most closely to the earlier generations for the following reasons.

In an expanding population, the earliest mutation strongly influences the final number of mutants. An early mutant carries forward to all descendant cells in an expanding mutant clone. If we start with the final cells and then look back through the cellular generations toward the original progenitor, the mutation with the most extreme time from the end toward the beginning tends to dominate the final mutant number.

The extreme value of a temporal extent often has a Gumbel distribution. In this case, once the mutation arises, it increases multiplicatively by cell division to affect the final mutation count. Substituting the extreme Gumbel time for its multiplicative consequence provides a common way to observe a Fréchet probability pattern.

Prior mathematical work also supports the Fréchet approximation. Kessler and Levine ^10^ showed that the Luria–Delbrück distribution converges to a Landau distribution for large *Nu*, in which the Landau distribution is a special case of the Lévy *α*-stable distribution. However, the Landau distribution does not have a closed-form expression for its probability or cumulative distribution functions.

Separately, Simon ^11^ showed the close match between the Lévy *α*-stable distribution and the Fréchet distribution. That match of a Lévy distribution to the Fréchet distribution had not previously been associated with the Luria–Delbrück distribution. The Fréchet parameterization in this article provides a simple expression that can be used to develop further theory and applications of the Luria–Delbrück process.

## Data Availability

The input data for calculating the empirical Luria–Delbrück CDF: Zenodo: Empirical CDF for Luria–Delbrück distribution from rSalvador package. https://doi.org/10.5281/zenodo.7075655 ^9^.

## Software availability

The Julia software code used to produce Figure 1:

Source code available from: https://github.com/evolbio/FrechetLD

Archived source code at time of publication: https://doi.org/10.5281/zenodo.7255050 ^8^

License: MIT
