# Supplementary material for: The number of neutral mutants in an expanding Luria-Delbrück population is approximately Fréchet
Source: F1000Res. 2023 Mar 3;11:1254. Originally published 2022 Nov 4. [Version 2] doi: 10.12688/f1000research.127469.2 (PMC9945811; doi:10.12688/f1000research.127469.2)
Supplement: Peer review history

# Peer review: Reviewer response for version 1

**DOI:** 10.5256/f1000research.139979.r162863

**Published:** 2023-02-20

**Authors:** Pavol Bokes

**Affiliations:** 1 Department of Applied Mathematics and Statistics, Comenius University, Bratislava, Slovakia

The paper compares the empirical (simulational) Luria-Delbruck mutant-number distribution to a Frechet distribution. The advantage of the Frechet distribution over other known approximations, e.g. the skewed alpha-stable distributions, is that it possesses a closed-form cumulative distribution function (cdf), see Equation (1). The parameters alpha, s, and beta of the Frechet distribution are set by the author as specific functions of the population-wide mutation rate N*u. Figure 1 visually demonstrates a solid agreement between the Frechet distribution and the empirical Luria-Delbruck distribution.

 Similarly to right-skewed alpha-stable distributions, the Frechet distribution has a heavy right tail and a light left tail (for z < beta the density is zero). It follows from (1) that the complementary cdf decays as 1/z^alpha, where alpha has been set by the author to exp(1)/2. In addition to the log-linear plots of Figure 1, it would be interesting to look closer at the power laws of the theoretical and empirical complementary cdfs e.g. using a log-log plot.

 Overall, the paper is well written, presents sound ideas, and develops an interesting approximation to the Luria-Delbruck mutant-number distribution.

Is the work clearly and accurately presented and does it cite the current literature?

Yes

If applicable, is the statistical analysis and its interpretation appropriate?

Yes

Are all the source data underlying the results available to ensure full reproducibility?

Yes

Is the study design appropriate and is the work technically sound?

Yes

Are the conclusions drawn adequately supported by the results?

Yes

Are sufficient details of methods and analysis provided to allow replication by others?

Yes

Reviewer Expertise:

Mathematical biology, stochastic modelling, gene expression, differential equations

I confirm that I have read this submission and believe that I have an appropriate level of expertise to confirm that it is of an acceptable scientific standard.

# Response

**Published:** 2023-02-20

**Authors:** Steven Frank

**Affiliations:** University of California, Irvine, USA

Thank you, I appreciate these comments. I agree that looking closely at the upper tail in a log-log plot would provide additional insight about the frequency of rare but potentially important events. However, to achieve a good computational estimate for the true cdf of the assumed process would require some new analyses to obtain precise estimates of the numerical error of the computation. That error could potentially be significant for the very rare upper tail events that would be the focus of such analysis. As applications arise that require a good estimate of the upper tail, the analyses and calculations would be a worthwhile new project.

# Peer review: Reviewer response for version 1

**DOI:** 10.5256/f1000research.139979.r155112

**Published:** 2022-11-23

**Authors:** Qi Zheng

**Affiliations:** 1 Department of Epidemiology and Biostatistics, Texas A&M University School of Public Health, College Station, TX, USA

In his brief report the author presents an interesting approximation of the Luria-Delbruck distribution, which microbiologists use to help determine microbial mutation rates in the laboratory. Specifically, equation (1) in the brief report is an approximation of the cumulative probability. If  denotes the probability of  mutants, the author implicitly defines the cumulative probability**  *__as*  *.*

 The author's key finding is that , where  is defined by equation (1) in the brief report. Note that the approximation in (1) is valid for any . However, as pointed out by the author, the approximation works well only for values of  that are noticeably larger than . I have conducted a number of computer experiments and confirmed the numerical results in the brief report. The approximation is theoretically interesting, and it may stimulate further theoretical developments. Thus, the paper merits indexing.

 I have a minor comment. There appears to be a typo in equation (1) in the brief report. If Prob( ) is changed to Prob( ), the correlative change in the definition of  will make  conform to the accepted definition of the cumulative probability. (That is, ) More importantly, this may make the approximation more accurate for small . Consider the case   (The symbol  here is the same as the symbol  in the brief report). Table 1 shows results obtained by using the revised definition, while Table 1A shows corresponding results obtained by using the original definition. In both tables, "error" refers to the following quantity: 

Is the work clearly and accurately presented and does it cite the current literature?

Yes

If applicable, is the statistical analysis and its interpretation appropriate?

Not applicable

Are all the source data underlying the results available to ensure full reproducibility?

Yes

Is the study design appropriate and is the work technically sound?

Yes

Are the conclusions drawn adequately supported by the results?

Yes

Are sufficient details of methods and analysis provided to allow replication by others?

Yes

Reviewer Expertise:

NA

I confirm that I have read this submission and believe that I have an appropriate level of expertise to confirm that it is of an acceptable scientific standard.

# Response

**Published:** 2023-02-20

**Authors:** Steven Frank

**Affiliations:** University of California, Irvine, USA

Thank you for the careful reading. With regard to the comment about m < z versus m <= z, the calculations to make figure 1 used m <= z for the empirical distribution, as recommended by the reviewer. For the theoretical continuous Frechet the numerical values are the same

 for the two cases. However, I agree that the notation in the original version of the manuscript is misleading. I will post a revised version that uses m <= z, as recommended.
