# Supplementary material for: The number of neutral mutants in an expanding Luria-Delbrück population is approximately Fréchet
Source: F1000Res. 2022 Nov 4;11:1254. [Version 1] doi: 10.12688/f1000research.127469.1 (PMC9945811; doi:10.12688/f1000research.127469.1)
Supplement: Peer review history

# Response

**Published:** 2023-02-20

**Authors:** Steven Frank

**Affiliations:** Department of Ecology & Evolutionary Biology, University of California, Irvine, USA

Thank you, I appreciate these comments. I agree that looking closely at the upper tail in a log-log plot would provide additional insight about the frequency of rare but potentially important events. However, to achieve a good computational estimate for the true cdf of the assumed process would require some new analyses to obtain precise estimates of the numerical error of the computation. That error could potentially be significant for the very rare upper tail events that would be the focus of such analysis. As applications arise that require a good estimate of the upper tail, the analyses and calculations would be a worthwhile new project.

# Response

**Published:** 2023-02-20

**Authors:** Steven Frank

**Affiliations:** Department of Ecology & Evolutionary Biology, University of California, Irvine, USA

Thank you for the careful reading. With regard to the comment about m < z versus m <= z, the calculations to make figure 1 used m <= z for the empirical distribution, as recommended by the reviewer. For the theoretical continuous Frechet the numerical values are the same

 for the two cases. However, I agree that the notation in the original version of the manuscript is misleading. I will post a revised version that uses m <= z, as recommended.
